# Decreased peripheral perfusion measured by perfusion index is a novel indicator for cardiovascular death in patients with type 2 diabetes and established cardiovascular disease

**DOI:** 10.1038/s41598-021-81702-w

**Published:** 2021-01-22

**Authors:** Hiroshi Okada, Muhei Tanaka, Takashi Yasuda, Yuki Okada, Hisahiro Norikae, Tetsuya Fujita, Takashi Nishi, Hirokazu Oyamada, Tetsuro Yamane, Michiaki Fukui

**Affiliations:** 1 Department of Diabetes and Endocrinology, Matsushita Memorial Hospital, 5-55 Sotojima-cho, Moriguchi, 570-8540 Japan; 2 Department of Endocrinology and Metabolism, Graduate School of Medical Science, Kyoto Prefectural University of Medicine, Kyoto, Japan; 3 Department of Nephrology, Matsushita Memorial Hospital, Moriguchi, Japan; 4 Department of General Affairs, Matsushita Memorial Hospital, Moriguchi, Japan; 5 Department of Gastroenterology, Matsushita Memorial Hospital, Moriguchi, Japan; 6 Department of Surgery, Matsushita Memorial Hospital, Moriguchi, Japan

**Keywords:** Cardiology, Diseases, Endocrinology

## Abstract

Cardiovascular disease (CVD) is still the major cause of mortality in patients with type 2 diabetes. Despite of recent therapies, mortality and resources spent on healthcare due to CVD is still important problem. Thus, appropriate markers are needed to predict poor outcomes. Therefore, we investigated the role of peripheral perfusion as an indicator for cardiovascular death in patients with type 2 diabetes and established CVD. This retrospective cohort study included 1080 patients with type 2 diabetes and history of CVD recruited from the outpatient clinic at Matsushita Memorial Hospital in Osaka, Japan. Peripheral perfusion is assessed using the perfusion index (PI), which represents the level of circulation through peripheral tissues. The median age and PI values were 74 years (range: 67–79 years) and 2.6% (range: 1.1–4.3%), respectively. During follow-up duration, 60 patients died due to CVD. The adjusted Cox regression analysis demonstrated that the risk of developing cardiovascular death was higher in the first quartile (Hazard ratio, 6.23; 95% CI, 2.28 to 22.12) or second quartile (Hazard ratio, 3.04; 95% CI, 1.46 to 6.85) of PI than that in the highest quartile (fourth quartile) of PI. PI (per 1% decrease) was associated with the development of cardiovascular death (Hazard ratio, 1.39; 95% CI, 1.16 to 1.68). PI could be a novel indicator of cardiovascular death in patients with type 2 diabetes and established CVD.

## Introduction

Cardiovascular disease (CVD) is the primary cause of mortality and morbidity in patients with type 2 diabetes, and several risk factors including smoking, hypertension and dyslipidemia have been shown to accelerate the progression of CVD^1,2^. Despite of advances of diabetic therapies, there are large residual risks of CVD in patients with type 2 diabetes. Previous studies have reported the signification of peripheral flow in the early phase of critical illnesses^3–6^. It has been suggested that microcirculatory alterations are stronger predictors of poor outcome than hemodynamic flow^5^. The peripheral perfusion index (PI) is the ratio of pulsatile blood flow to non-pulsatile blood flow in the monitored tissue and has been shown to reflect changes in peripheral perfusion^7–9^. A PI of 1.4 has been suggested to be correlated with low peripheral perfusion in critically ill patients^7^. Moreover, low value of PI was associated with adverse outcomes in the early phase of critical illnesses^9,10^. However, there are no studies which reported the association between PI and the development of cardiovascular death in patients with type 2 diabetes in clinical care setting. Therefore, we aimed to assess the association between PI and the development of cardiovascular death and the association between PI and cardiovascular death or recurrent cardiovascular events in patients with type 2 diabetes and established CVD.

## Results

The characteristics of all 1080 patients enrolled in this study are shown in Table 1. The average duration of follow up was 871 ± 265 days. The median age and PI values were 74 years (range: 67–79 years) and 2.6% (range: 1.1–4.3%), respectively. Sixty patients died due to cardiovascular death during study period (Quartile 1, 2, 3 and 4; 32,14,10 and 4, respectively). Cardiovascular death included 11 myocardial infarction, 44 heart failure, and 5 stroke during study period. One hundred and ninety-six patients developed the recurrent cardiovascular events during study period (Quartile 1,2,3 and 4; 86, 48, 37 and 25, respectively). Four patients developed myocardial infarction, 16 patients developed stroke, 120 patients received revascularization for angina and 56 patients received revascularization for peripheral artery disease (PAD).

**Table 1:** Characteristics of patients.

n (male/female)	1080 (774/306)
Age (years)	74 (67–79)
Duration of diabetes (years)	7.5 (4–12)
Body mass index (kg/m^2^)	24.6 (22.1–27.4)
Systolic blood pressure (mmHg)	131 (117–143)
Diastolic blood pressure (mmHg)	72 (61.3–82)
Heart rate (beat per minutes)	75 (66–86)
Hemoglobin A1c (%)	6.8 (6.3–7.7)
Total cholesterol (mg/dl)	170 (146–203)
Low-density lipoprotein cholesterol (mg/dl)	94.2 (68.2–121.15)
Triglycerides (mg/dl)	116 (87–166)
Uric acid (mg/dl)	5.5 (4.4–6.8)
Creatinine (mg/dl)	0.96 (0.79–1.40)
Smoking status (never/past/recent)	448/234/398
Hypertension (−/ +)	298/782
History of CVD (angina or myocardial infarction/stroke/peripheral artery disease)	765/298/161
Previous revascularization history (−/ +)	595/485
Perfusion index (%)	2.6 (1.1–4.3)
Anti-platelet therapies (−/ +)	153/927
Renin-angiotensin system inhibitor (−/ +)	484/596
Glucagon-like peptide-1 agonists (−/ +)	984/116
Sodium-glucose cotransporter 2 inhibitor (−/ +)	983/97
Statin (−/ +)	476/604

Data are expressed as the median (interquartile range) or absolute number.

CVD, cardiovascular disease.

Patients with the development of cardiovascular death were older (*P* = 0.03) than those without at baseline. Their average SBP (*P* = 0.02) and serum creatinine (*P* < 0.0001) were higher than those without at baseline. Patients with the development of cardiovascular death had longer duration of diabetes than that without at baseline (*P* = 0.02). PI or total cholesterol was lower at baseline in patients with the development of cardiovascular death than that without (*P* < 0.0001 or *P* = 0.0007). We found negative correlation between PI and age (*r* = − 0.15, *P* < 0.0001), heart rate (*r* = − 0.15, *P* < 0.0001) or serum creatinine (*r* = − 0.14, *P* < 0.0001). We found positive correlation between PI and body mass index (BMI) (*r* = 0.11, *P* = 0.0002).

Table 2 reports characteristics of the study participants at the baseline according to quartiles of PI. The proportion of male was higher in highest quartile of PI (quartile 4) than those in in the other quartiles. Participants in the highest quartile of PI (quartile 4) was younger, had higher BMI and had lower heart rate than those in the other quartile. The serum creatinine level in the lowest quartile of PI (quartile 1) was higher than those in the other quartile. Tables 3 and 4 shows the results of the Cox regression analyses. The unadjusted Cox regression analysis revealed that the risk for the development of cardiovascular death was higher in quartile 1 or 2 than that in quartile 4 (reference). Additionally, the unadjusted Cox regression analyses demonstrated that age (Hazard ratio, 1.04; 95% CI, 1.01 to 1.07), duration of diabetes (Hazard ratio, 1.04; 95% CI, 1.004 to 1.07), average SBP (Hazard ratio, 1.01; 95% CI, 1.00 to 1.03), total cholesterol (Hazard ratio, 0.99; 95% CI, 0.98 to 0.997) or serum creatinine (Hazard ratio, 1.16; 95% CI, 1.08 to 1.24) was associated with an increased hazard of the development of cardiovascular death.

**Table 2:** Characteristics at baseline according to quartiles of perfusion index.

	Quartile 1	Quartile 2	Quartile 3	Quartile 4	
Perfusion index (%)	≤ 1.10	1.11–2.60	2.61–4.30	≥ 4.31	*P*-value
n	278	284	254	264	–
Sex (male/female)	188/90	196/88	176/78	214/50	0.002
Age (y)	75(68–80)	74.5(67–81)	74(68–78)	71(62–77)	< .0001
Duration of diabetes (y)	8 (4–12)	7 (4–12)	8 (4–13)	6 (3–12)	0.14
BMI (kg/m^2^)	24.0 (21.4–27.8)	24.6 (21.6–27.5)	24.4 (22.1–27.4)	25.1 (23.1–27.3)	0.008
Average SBP (mmHg)	133 (117–147)	131 (118–143)	130 (116–141)	129 (117–141)	0.33
Heart rate (beat per minutes)	76 (68–90)	77.5 (68–86)	74 (67–85)	72.5 (61–84)	0.0002
Hemoglobin A1c (%)	6.8 (6.2–7.9)	7 (6.4–7.8)	6.6 (6.2–7.4)	6.9 (6.3–7.8)	0.11
T-CHO (mg/dl)	168 (146–199)	170 (143–204)	172 (149–207)	167 (150–201)	0.27
Triglycerides (mg/dl)	114 (89–149)	115 (83–170)	117 (88–154)	117 (84–181)	0.33
Uric acid (mg/dl)	5.6 (4.3–6.9)	5.4 (4.3–6.7)	5.4 (4.4–6.7)	5.7 (4.6–6.8)	0.41
Creatinine (mg/dl)	1.07 (0.84–3.24)	0.95 (0.76–1.26)	0.95 (0.79–1.25)	0.92 (0.75–1.27)	< .0001
Smoking (%)		0.003			
Never	45.3	40.1	44.1	36.4	
Previous	25.2	23.2	14.7	23.5	
Current	29.5	36.6	41.3	40.2	
PI (%)	0.56 (0.25–0.84)	2.0 (1.5–2.2)	3.3 (3.0–3.8)	5.8 (5.0–7.0)	< .0001

Data are expressed as the median (interquartile range) or absolute number.

BMI, body mass index; SBP, systolic blood pressure; T-CHO, total cholesterol.

*P* values are from chi-square tests (for categorical data) or from ANOVA (for continuous data).

**Table 3:** Unadjusted hazard ratios and multivariate adjusted hazard ratios for cardiovascular death.

	Quartile of perfusion index				*P*
	Quartile 1	Quartile 2	Quartile 3	Quartile 4	–
PI (%)	≤ 1.10	1.11–2.60	2.61–4.30	≥ 4.31	–
Crude	8.31 (3.30–27.92)	3.43 (1.23–12.09)	2.60 (0.87–9.47)	1 (reference)	< .0001
Model 1	5.65 (2.17–19.34)	2.70 (1.35–5.88)	2.01 (1.07–3.94)	1 (reference)	0.001
Model 2	6.23 (2.28–22.12)	3.04 (1.46–6.85)	2.43 (1.20–5.22)	1 (reference)	0.001

PI, perfusion index.

Model 1 is adjusted for sex, age, body mass index, heart rate, creatinine and smoking status. Model 2 includes all variables in Model 1 plus duration of diabetes, systolic blood pressure, hemoglobin A1c, total cholesterol, anti-platelet therapies, renin-angiotensin system inhibitor, glucagon-like peptide-1 agonists, sodium-glucose cotransporter 2 inhibitor and statin.

**Table 4:** Unadjusted hazard ratios and multivariate adjusted hazard ratios for cardiovascular death or recurrent cardiovascular events.

	Quartile of perfusion index				*P*
	Quartile 1	Quartile 2	Quartile 3	Quartile 4	–
PI (%)	≤ 1.10	1.11–2.60	2.61–4.30	≥ 4.31	–
Crude	3.40 (2.27–5.27)	1.73 (1.12–2.76)	1.46 (0.92–2.36)	1 (reference)	< .0001
Model 1	3.34 (2.19–5.24)	1.64 (1.04–2.63)	1.37 (0.86–2.24)	1 (reference)	< .001
Model 2	3.05 (1.97–4.84)	1.36 (0.86–2.21)	1.04 (0.64–1.72)	1 (reference)	< .0001

PI, perfusion index.

Model 1 is adjusted for sex, age, body mass index, heart rate, creatinine and smoking status. Model 2 includes all variables in Model 1 plus duration of diabetes, systolic blood pressure, hemoglobin A1c, total cholesterol, anti-platelet therapies, renin-angiotensin system inhibitor, glucagon-like peptide-1 agonists, sodium-glucose cotransporter 2 inhibitor and statin.

**Figure 1:**
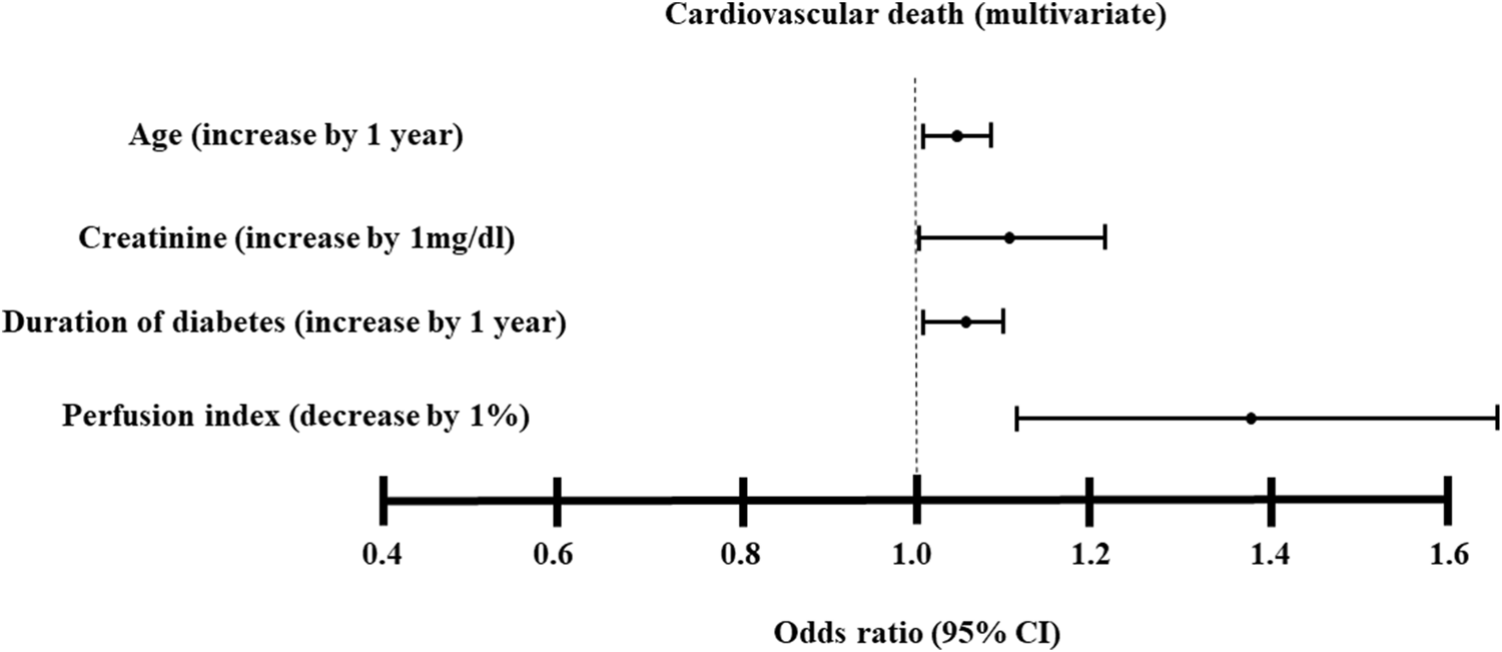
Predictors of cardiovascular death (multivariate analyses). The multivariate Cox proportional hazards regression models were done the following variables: sex, age, duration of diabetes, body mass index, systolic blood pressure, heart rate, hemoglobin A1c, total cholesterol, creatinine, smoking status, anti-platelet therapies, renin-angiotensin system inhibitor, glucagon-like peptide-1 agonists, sodium-glucose cotransporter 2 inhibitor and statin (model 2). Variables that remained significantly associated with cardiovascular death are presented.

**Figure 2:**
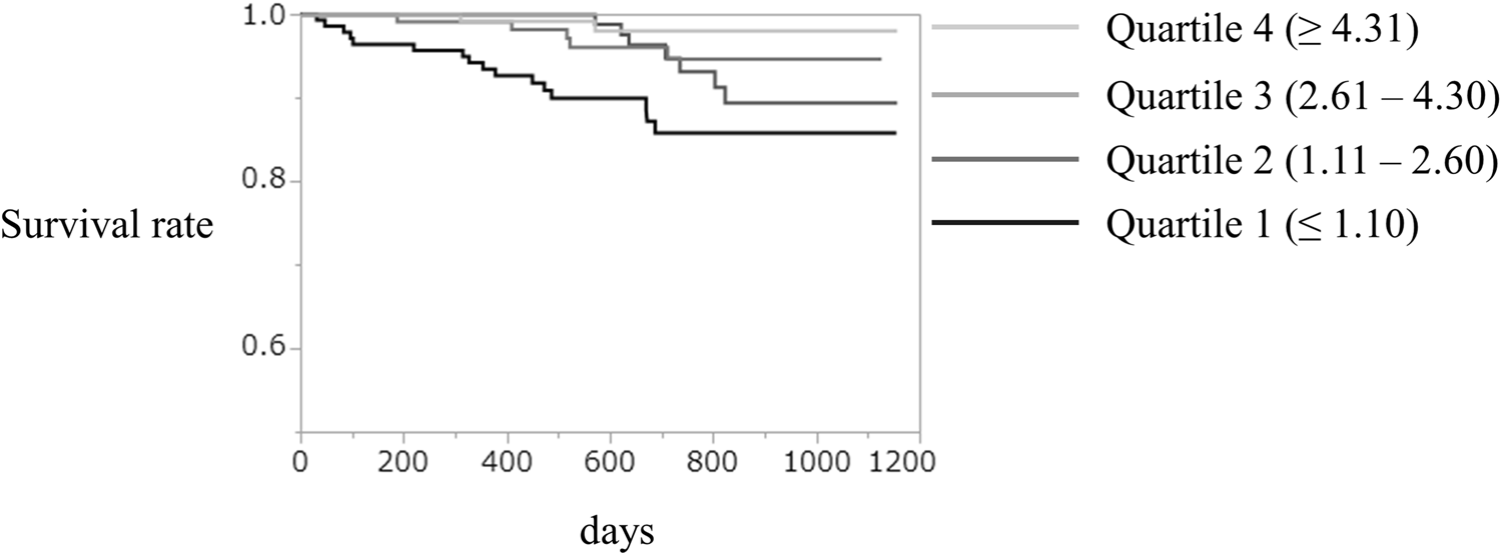
The Kaplan–Meier survival curve stratified according to perfusion index at baseline.

The adjusted Cox regression analysis (Model 1 and 2) revealed that the risk for the development of cardiovascular death was higher in quartile 1, 2 or 3 than that in quartile 4 (reference). Moreover, the adjusted Cox regression analysis revealed that PI (per 1% decrease) was associated with the development of cardiovascular death (Hazard ratio, 1.39; 95% CI, 1.16 to 1.68) and age (Hazard ratio, 1.04; 95% CI, 1.003 to 1.08), creatinine (Hazard ratio, 1.11; 95% CI, 1.001 to 1.22) or duration of diabetes (Hazard ratio, 1.06; 95% CI, 1.02 to 1.11) remained significant indicators of cardiovascular death (Fig. 1). Times to event for cardiovascular death by PI category are also illustrated with Kaplan–Meier curves (Fig. 2). The adjusted Cox regression analysis (Model 2) revealed that the risk for the development of cardiovascular death or recurrent cardiovascular events was higher in quartile 1 than that in quartile 4 (reference). We assessed ankle brachial index (ABI) data among 495 patients. PI was positively correlated with ABI (*r* = 0.26, *P* < 0.0001). The adjusted Cox regression analysis (model 2) revealed that ABI (per 0.1 decrease) was associated with the development of cardiovascular death (Hazard ratio, 1.70; 95% CI, 1.1 to 2.66). There were 136 patients with ABI ≤ 0.9. Moreover, the adjusted Cox regression analysis revealed that PI (per 1% decrease) was associated with the development of cardiovascular death (Hazard ratio, 1.49; 95% CI, 1.30 to 1.70) among patients without ABI ≤ 0.9 (n = 359).

## Discussion

The major finding of our study is that PI, which represents peripheral perfusion is a novel indicator for cardiovascular death in patients with type 2 diabetes and established CVD.

Blood flow is diverted from peripheral tissues to vital organs during circulatory shock. Peripheral tissues are considered as the first tissue bed to be sacrificed in circulatory shock^11,12^. Despite the normalization of hemodynamic flow with delivery of fluids and vasoactive compounds during circulatory shock, some patients enter a phase where decreased peripheral perfusion becomes more evident^10^. It has been reported that persisting peripheral perfusion dysfunction, despite normalization of hemodynamic flow, is associated with unfavorable outcomes^6,10,13–16^. Lima et al.^10^ have found that patients who failed to normalize peripheral perfusion calculated by PI in the early phase of critical illnesses had a worse outcome. Moreover, He et al.^9^ showed that the PI could predict mortality with similar accuracy to arterial lactate levels and found that the cut off value of the PI was ≤ 0.2 for predicting mortality in the early phase of critical illnesses, resulting in a sensitivity of 65% and a specificity of 92.3%. Taken together, decreased peripheral perfusion was associated with poor outcomes in the early phase of critical illnesses. On the other hand, in general care setting, the significance of peripheral perfusion dysfunction has not been well known. We have some patients who have gap between hemodynamic flow and peripheral perfusion in general care setting. Although peripheral perfusion could be associated with the severity of cardiogenic shock in the early phase of critical illnesses^4^, it might be associated with nutritional status, atherosclerotic CVD and sympathetic nerve activity in general care setting. Indeed, this study shows that PI correlated positively with BMI and correlated negatively with age, which suggest that peripheral perfusion could be affected by nutrition status. We have recently reported that PI is associated with ABI^17^, which suggest that peripheral perfusion could be affected by the presence of atherosclerotic CVD. Moreover, this study shows that PI correlated negatively with heart rate, which suggest that peripheral perfusion could be affected by sympathetic nerve activity. It is well known that heart rate is inversely related to the lifespan^18^. The patients with low value of PI might have poor nutritional status, advanced atherosclerotic CVD and high sympathetic nerve activity. In our study, PI is the associated with cardiovascular death in patients with type 2 diabetes and established CVD after adjustment for age, BMI and heart rate.

PI might have an important role for the prevention of cardiovascular death in patients with type 2 diabetes. There are some reports indicating improvement in microcirculatory dysfunction with fluid administration and adrenergic agonist in the early phase of critical illnesses^19–21^. However, interventional methods to improve PI have not been established. Then, patients with low value of PI might require aggressive lifestyle modifications and medication to lower blood glucose and blood pressure.

ABI is known as the gold standard for PAD which is associated with CVD. In our study, ABI was associated with the development of cardiovascular death and PI was positively associated with ABI. However, PI was associated with cardiovascular death in patients without ABI ≤ 0.9. PI takes only a few minutes to complete and is very simple. Multiple steps are not required and the patients will not lose control of their mobility while wearing the sensors. PI equipment is less expensive than the ABI equipment. The PI could add clinical benefit and be available for the patients with type 2 diabetes in the health care system.

There are several limitations. It may be unclear if our results are applicable to patients of other ethnicities because only Japanese patients were targeted in this study. Because most of the subjects of this study were elderly patients, it may be unclear if our results are generalized to all range of age in patients with diabetes. Moreover, because the number of patients was not large, large investigations with patients of various ethnicities and range of age would be needed to prevent cardiovascular death in patients with type 2 diabetes.

To the best of our knowledge, this is the first study to investigate if PI could be an indicator for cardiovascular death in patients with type 2 diabetes and established CVD. We focus from monitoring hemodynamic flow to monitoring peripheral perfusion in the early phase of critical illnesses and we might also need to consider the alternation of peripheral perfusion in clinical care setting. In conclusion, PI could be a valuable indicator of cardiovascular death in patients with type 2 diabetes and established CVD.

## Materials and methods

### Ethics

This study was approved by local research ethics committee of Matsushita Memorial Hospital and has been conducted in accordance with the ethical principles of the Declaration of Helsinki. Written informed consent was obtained from all of the patients.

### Patients and data collection

We performed a retrospective cohort study in 1080 patients recruited from the outpatient clinic at Matsushita Memorial Hospital in Osaka, Japan, between September 2015 and September 2016. Patients were eligible if they had type 2 diabetes and history of CVD. We collected the information on smoking status and past history using the self-administered questionnaire. All the data were retrieved from the database. Fasting blood samples were obtained in the morning. Total serum cholesterol and triglyceride concentrations were assessed using standard enzymatic methods. Low-density lipoprotein (LDL) cholesterol was calculated using Friedewald formula: LDL Cholesterol = Total Cholesterol—High-density lipoprotein Cholesterol—(Triglycerides / 5)^22^.

Diabetes was diagnosed based on the Report of the Expert Committee on the Diagnosis and Classification of Diabetes Mellitus^23^. Hypertension was defined when the patient’s systolic blood pressure was greater than 140 mmHg, their diastolic blood pressure was greater than 90 mmHg, and/or the patient was prescribed any antihypertensive medications. Patients were classified as nonsmokers, past smokers, or current smokers based on a self-administered questionnaire. CVD was defined as angina, myocardial infarction, stroke or PAD based on a self-administered questionnaire. Cardiovascular death was defined as that due to myocardial infarction, heart failure, or stroke. Recurrent cardiovascular events were defined as myocardial infarction, stroke or revascularization for angina or PAD. Patients for whom PI measurement could not be obtained were excluded from this study; those with implanted cardiac pacemakers; arrhythmia, such as paroxysmal atrial fibrillation; or amputations of any part of the lower extremities were also excluded from the study. Patients with malignancy at baseline were excluded.

### Technique for determining PI

PI was measured using a Masimo SET Radical-7 (Masimo Corporation, Irvine, CA) instrument. The patients were placed in the supine position. A Masimo pulse oximeter probe was positioned on each toe and connected to the Masimo SET Radical-7 machine. The patients were asked to rest for 5 min at the beginning of the procedure. PI was then recorded three times: at 20, 40, and 60 s, after the 5-min rest period. The average of the three values was calculated and used as the reference value. The Masimo SET Radical-7 calculates PI as the ratio between the pulsatile and non-pulsatile components of the light reaching a light-sensitive cell of the pulse oximetry probe. The reliability and reproducibility of PI have been reported elsewhere^17,24^. After PI was determined bilaterally, the lower value was considered as a representative for each subject.

### Statistical analysis

We categorized the participants according to the quartiles of their PI to evaluate the association between characteristics at baseline and PI: ≤ 1.10, 1.11–2.60, 2.61–4.30, and ≥ 4.31%. We reported the means or percentages for each quartile, and associations were assessed by ANOVA or chi-square test^24^. The differences of general characteristics at baseline according to the development of cardiovascular death at follow up were assessed by t-test or chi-square test^25^. The relationships between PI and other variables were assessed by Spearman’s rank correlation analyses^25^. Log transformation was carried out before performing Spearman’s rank correlation analyses because triglycerides showed skewed distributions. The association between PI or ABI and the development of cardiovascular death or composite development of cardiovascular death or recurrent cardiovascular events was analyzed in Cox proportional hazards regression models^25^. An unadjusted and a multivariate model were used for the estimation of association between PI and the development of cardiovascular death or composite development of cardiovascular death or recurrent cardiovascular events^25^. Statistically significant variables in univariate analysis and those known to be related factors for the development of cardiovascular death were included as covariates in the multivariate model were. Model 1 is adjusted for sex, age, body mass index, heart rate, creatinine and smoking status. Model 2 was adjusted for all variables in Model 1 plus duration of diabetes, systolic blood pressure (SBP), hemoglobin A1c, total cholesterol, anti-platelet therapies, renin-angiotensin system inhibitor, glucagon-like peptide-1 agonists, sodium-glucose cotransporter 2 inhibitor and statin. Time-to-event distributions in the categories were summarized with Kaplan–Meier curves.

## Data Availability

The datasets generated during the current study are available from the corresponding author on reasonable request**.**

## References

[1] Isomaa B (2001). Cardiovascular morbidity and mortality associated with the metabolic syndrome. Diabetes Care.

[2] Multiple Risk Factor Intervention Trial Research Group (1982). Multiple risk factor intervention trial. Risk factor changes and mortality results. JAMA.

[3] Lima A, Bakker J (2005). Noninvasive monitoring of peripheral perfusion. Intensive Care Med..

[4] den Uil CA (2010). Impaired microcirculation predicts poor outcome of patients with acute myocardial infarction complicated by cardiogenic shock. Eur. Heart J..

[5] De Backer D (2013). Microcirculatory alterations in patients with severe sepsis: Impact of time of assessment and relationship with outcome. Crit. Care Med..

[6] Trzeciak S (2008). Microcirculatory Alterations in Resuscitation and Shock (MARS) Investigators: early increases in microcirculatory perfusion during protocol-directed resuscitation are associated with reduced multi-organ failure at 24 h in patients with sepsis. Intensive Care Med..

[7] Lima A, Beelen P, Bakker J (2002). Use of a peripheral perfusion index derived from the pulse oximetry signal as a noninvasive indicator of perfusion. Crit. Care Med..

[8] Galvin EM (2006). Peripheral flow index is a reliable and early indicator of regional block success. Anesth. Analg..

[9] He H, Liu D, Long Y, Wang X (2013). The peripheral perfusion index and transcutaneous oxygen challenge test are predictive of mortality in septic patients after resuscitation. Crit. Care.

[10] Lima A, Bakker J (2014). Clinical monitoring of peripheral perfusion: there is more to learn. Crit. Care.

[11] Chien LC, Lu KJ, Wo CC, Shoemaker WC (2007). Hemodynamic patterns preceding circulatory deterioration and death after trauma. J. Trauma.

[12] Poeze M, Solberg BC, Greve JW, Ramsay G (2005). Monitoring global volumerelated hemodynamic or regional variables after initial resuscitation: what is a better predictor of outcome in critically ill septic patients?. Crit. Care Med..

[13] Payen D (2009). Is thenar tissue hemoglobin oxygen saturation in septic shock related to macrohemodynamic variables and outcome?. Crit. Care.

[14] van Genderen ME, Lima A, Akkerhuis M, Bakker J, van Bommel J (2012). Persistent peripheral and microcirculatory perfusion alterations after out-of-hospital cardiac arrest are associated with poor survival. Crit. Care Med..

[15] Lima A, van Bommel J, Jansen TC, Ince C, Bakker J (2009). Low tissue oxygen saturation at the end of early goal-directed therapy is associated with worse outcome in critically ill patients. Crit. Care.

[16] Lima A, Jansen TC, van Bommel J, Ince C, Bakker J (2009). The prognostic value of the subjective assessment of peripheral perfusion in critically ill patients. Crit. Care Med..

[17] Okada H (2019). The perfusion index is a useful screening tool for peripheral artery disease. Heart Vessels.

[18] Zhang GQ, Zhang W (2009). Heart rate, lifespan, and mortality risk. Ageing Res. Rev..

[19] Morelli A (2010). Levosimendan for resuscitating the microcirculation in patients with septic shock: a randomized controlled study. Crit. Care.

[20] van Genderen M, Gommers D, Klijn E, Lima A, Bakker J, van Bommel J (2011). Postoperative sublingual microcirculatory derangement following esophagectomy is prevented with dobutamine. Clin. Hemorheol. Microcirc..

[21] Dubin A (2010). Comparison of 6% hydroxyethyl starch 130/0.4 and saline solution for resuscitation of the microcirculation during the early goal-directed therapy of septic patients. J. Crit. Care.

[22] Friedewald WT, Levy RI, Fredrickson DS (1972). Estimation of the concentration of low-density lipoprotein cholesterol in plasma, without use of the preparative ultracentrifuge. Clin. Chem..

[23] Expert Committee on the Diagnosis and Classification of Diabetes Mellitus (2003). Report of the expert committee on the diagnosis and classification of diabetes mellitus. Diabetes Care.

[24] Quinn CT, Raisis AL, Musk GC (2013). Evaluation of Masimo signal extraction technology pulse oximetry in anaesthetized pregnant sheep. Vet. Anaesth. Analg..

[25] Okada H (2014). Low serum bilirubin concentration is a novel risk factor for the development of albuminuria in patients with type 2 diabetes. Metabolism.

